# The Synthesis of the Core/Shell Structured Diamond/Akageneite Hybrid Particles with Enhanced Polishing Performance

**DOI:** 10.3390/ma10060673

**Published:** 2017-06-20

**Authors:** Jing Lu, Yongchao Xu, Dayu Zhang, Xipeng Xu

**Affiliations:** 1 Institute of Manufacturing Engineering, Huaqiao University, Xiamen 361021, China; beyondmyselfxyc@163.com (Y.X.); zdy130312@163.com (D.Z.); xpxu@hqu.edu.cn (X.X.); 2 MOE Engineering Research Center for Brittle Materials Machining, Huaqiao University, Xiamen 361021, China

**Keywords:** hybrid particle, isothermal hydrolyzing, sapphire, polishing performance

## Abstract

In this study, the synthesis of the core/shell structured diamond/akageneite hybrid particles was performed through one-step isothermal hydrolyzing. The hybrid particle was characterized by X-ray diffraction, field emission scanning electron microscopy, and Fourier transform infrared spectra. The test results overall reveal that the akageneite coating, phase β-FeO(OH), was uniformly coated onto the diamond surface. The polishing performance of the pristine diamond and hybrid particles for the sapphire substrate was evaluated respectively. The experimental results show that the hybrid particles exhibited improved polishing quality and prolonged effective processing time of polishing pad compared with diamond particles without compromising the material remove rate and surface roughness. The improved polishing behavior might be attributed to the β-FeOOH coating, which is conducive to less abrasive shedding and reducing the scratch depth.

## 1. Introduction

Sapphire, the most common substrate used in light emitting diodes (LEDs), exhibits excellent mechanical and optical properties and is widely used in a range of applications, such as optics, electronics, and temperature sensing, etc. [1,2,3,4,5,6]. In order to realize these applications based on sapphire, a smooth and planar surface without scratches and subsurface-damaged layers is required [7,8,9,10]. Currently, the main planarization machining to realize the precision requirement for sapphire substrate is still abrasive machining, especially at fine grain size. However, for the free abrasive polishing, such as chemical mechanical polishing (CMP), it is extremely difficult to polish the sapphire substrates to obtain high material remove rate (MRR) and perfect polished surface simultaneously. For the fixed abrasive polishing, the abrasive particles with agglomerate effect are difficult to be dispersed evenly in the binder when the size of the particle is small to a certain degree, which is difficult to obtain a high-quality surface [11,12]. So, the creation of nontraditional polishing tools has attracted considerable attention to process sapphire substrate.

Based on the principle of sol-gel, a semi-fixed abrasive polishing pad with diamond abrasive, called SG polishing pad, is an ideal tool that satisfies the processing demands of various kinds of hard and brittle materials [13,14,15]. However, the abrasives easily fall off from the SG polishing tool during process, leading to the low efficiency and short life of the polishing tool. Moreover, the scratch defect and mechanical damage are easily induced because the diamond particles are irregular in shape and possess sharp edges, corners, and apexes with high hardness.

Core/shell structures exhibit unique structure and special properties and thus can be used to efficiently change the mechanical properties of abrasives [16,17,18,19]. Akageneite features a tunnel structure and superior capacity in catalysts [20,21,22]. Akageneite also possesses high specific surface area and substantial –OH; as such, this mineral displays potential for promoting binding force with the matrix of polishing pad. Moreover, akageneite with low hardness value can reduce the mechanical damages caused by the sharp edges of diamond abrasives.

In this study, we described a novel route for preparing core/shell structured diamond/akageneite hybrid particle via one-step isothermal hydrolyzing to reduce the abrasive shedding and mechanical damage during polishing. In contrast to forced hydrolysis and other severe preparations which are always under the temperature above 90 °C [23], this isothermal hydrolyzing procedure under relatively low temperature (40 °C) is very slow and soft [24,25]. The formation of akageneite is prone to occur on the surface of diamond rather than in the solution. To elucidate their structure and composition, the hybrid particles were characterized by X-ray diffraction (XRD), field emission scanning electron microscope (FESEM), and Fourier transform infrared spectra (FTIR). Moreover, based on the SG polishing tool, the polishing performance of the pristine diamond and hybrid particles for sapphire substrate were evaluated respectively.

## 2. Experimental Section

### 2.1. Synthesis of Diamond/Akageneite Hybrid Particles

The hybrid particles were synthesized by one-step isothermal hydrolyzing method. The iron (III) chloride hexahydrate (AR) was purchased from Xilong Chemical Co., Ltd. (Guangzhou, China). Diamond abrasives with similar shape and an average primary size of 3 μm were produced by Element Six Trading CO., Ltd. (Shanghai, China). Solid FeCl_3_·6H_2_O was first dissolved in 400 mL of distilled water to produce 0.04 M solution under magnetic stirring. Then 0.5 g of diamond was added into the aqueous solution. After 5 min ultrasonic vibration, a yellow suspension with diamond abrasives homogeneously dispersed was obtained. The stable aqueous suspension was heated at 40 °C and stirred in a thermostatic water bath for 48 h to ensure the isothermal hydrolyzing of FeCl_3_. After that, the precipitate was further processed by centrifugal cleaning and drying. The mechanism for the formation of core-shell structural diamond/akageneite hybrid particle by the isothermal hydrolyzing of FeCl_3_ aqueous solution could be given as

FeCl_3_ + 3H_2_O → Fe(OH)_3_ + 3HCl (1)

Fe(OH)_3_ → β-FeOOH + H_2_O (2)

The XRD profile of the hybrid particle was examined by an X-ray diffractometer (X’ Pert PRO, PANalytical B.V., Almelo, The Netherlands) using Cu Kα radiation (λ = 1.54055 Å) with an accelerating voltage of 40 kV and a current of 40 mA. The morphology and microstructure of the samples were observed by a FESEM (S-4800, Hitachi Limited, Tokyo, Japan), FTIR measurement of hybrid particle was conducted using a FT-IR spectrometer (Thermo Scientific Nicolet iS10, Thermo Fisher Scientific, Waltham, MA, USA).

### 2.2. Polishing Setup and Process Control

The epi-ready sapphire wafers were purchased commercially. Sapphire wafer-oriented (0 0 0 1) plane with two-inch diameter after grinding was used. The original surface roughness (Ra) of the wafer after lapping is about 1.8 nm. Pristine diamond and hybrid particles were used as abrasives in SG polishing pad, respectively. The flexible matrix of SG polishing pad was made from sodium alginate (AGS), and the fabrication of the SG polishing pad mainly included mixing, screeding, gelling, and drying. A rotary-type polishing machine (AUTOPOL-1200S, Kejing, Shenyang, China) was used in the sapphire-polishing experiments. The pressure between the sample clamp and the polishing pad was 5 kg. The speed of workpiece/pad rotation was 60/120 rpm and the treatment time was 3 h. Deionized water was applied as coolant. After processing, the wafers were cleaned immediately with ethanol and deionized water under sonication. The surface roughness (Ra) and morphology of processed substrates were measured by 3D optical interferometry profiler (New View™ 7300, Zygo, Middlefield, OH, USA). For the testing of surface roughness polished by diamond abrasives, the deep scratches were avoided. The MRR as a function of processing time is calculated by

(3) $$MRR=\frac{10^{7}\times \Delta m}{\rho \times 2.54^{2}\times \pi \times t}$$

where Δ*m* (mg) is the mass loss of sapphire wafer after polishing, *t* (min) is the processing time, *ρ* is the sapphire wafer density (3.98 g/cm^3^).

## 3. Results and Discussion

**Figure 1:**
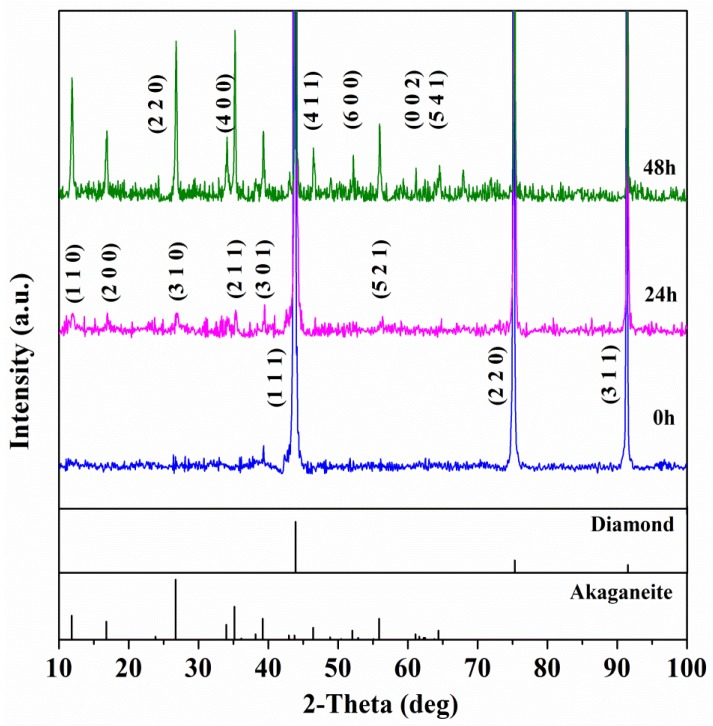
XRD patterns of diamond/akageneite hybrid particle with different reaction durations.

The XRD patterns of diamond/akageneite hybrid particle with different reaction durations are presented in Figure 1. The characteristic peaks at 2θ = 11.8°, 16.8°, 23.8°, 26.7°, 34.0°, 35.2°, 39.2°, 46.4°, 52.0°, 55.9°, 61.1° and 64.4° could be indexed as akageneite (PDF-34-1266). The characteristic peaks at 2θ = 43.9°, 75.3°, and 91.5° could be indexed as diamond (PDF-06-0675). All of the visible diffraction peaks match well with the feature peaks of akageneite and diamond, which were mixed with the diamond and akageneite phase. No third phase was observed. The diffraction intensity of the akageneite increased with its growth as the reaction time increased.

**Figure 2:**
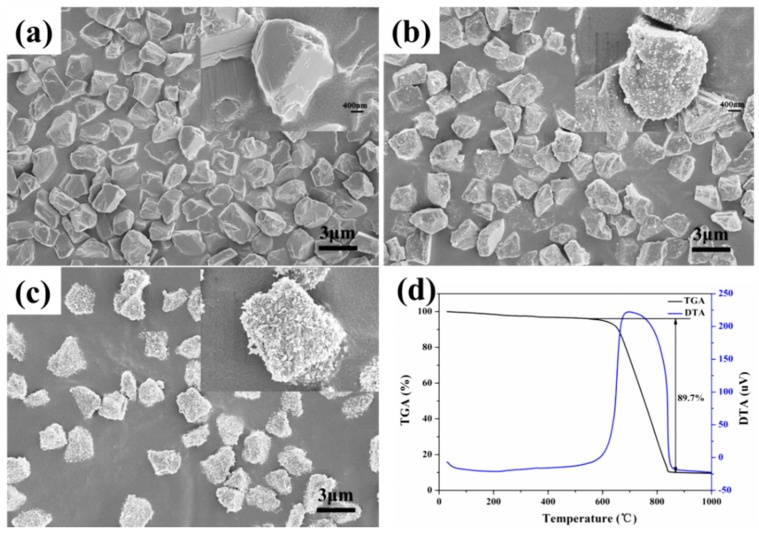
Morphologies of diamond/akageneite hybrid particles under different reaction durations: (**a**) 0 h; (**b**) 24 h; (**c**) 48 h; and (**d**) TGA-DTA curves of the hybrid particle under 48 h of reaction.

Figure 2a shows the FESEM image of pristine diamond particles. It can be seen that the pristine diamond particles are uniform in shape and the particle surface is clean. Figure 2b presents the status of nucleation and growth of β-FeOOH on the diamond particle surface. β-FeOOH continues to grow with the reaction until the formation of uniform spindle-shaped coating on the diamond particle surface, as shown in Figure 2c. Figure 2d shows the thermogravimetry-differential thermal analysis (TGA-DTA) curves of the hybrid particle under 48 h of reaction. As shown in Figure 2d, the endothermic peak at about 700 °C corresponds to the 89.7% weight loss on thermogravimetry (TG) curve, which is attributed to the oxidation of diamond. The weight loss of diamond on TG curve indicates that the weight percent of the coating is around 10%.

**Figure 3:**
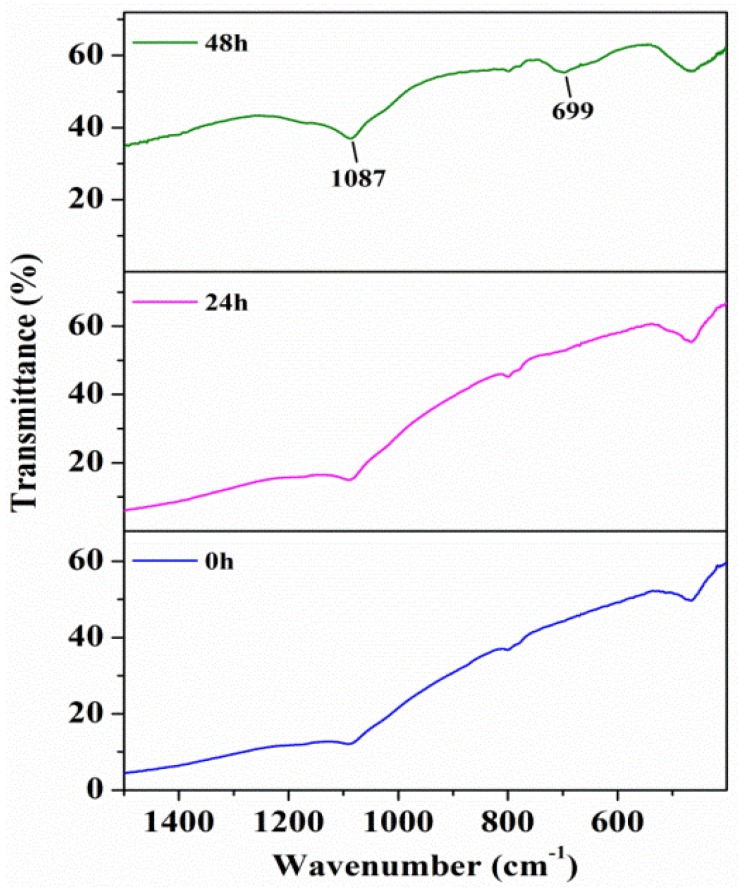
FTIR spectra of diamond/akageneite hybrid particles with different reaction durations.

The FTIR spectra of hybrid particles with different reaction durations are shown in Figure 3. The absorption peaks at 1087 and 699 cm^−1^ are attributed to the –OH stretching and Fe–O vibrational modes of β-FeOOH [23,26,27], which is benefit for the interface bonding. Compared with the FTIR spectra of pristine diamond particles, the absorption peaks of β-FeOOH gradually increased with the increasing reaction time.

**Figure 4:**
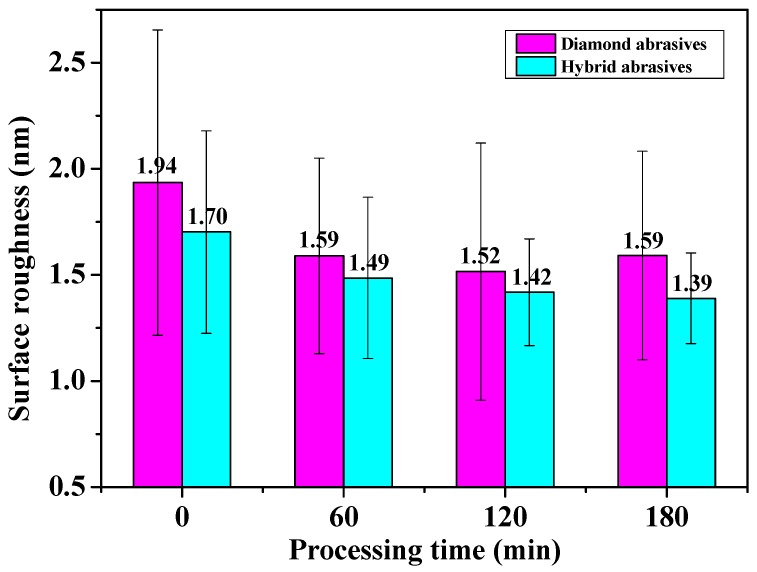
Roughness of sapphire substrate polished by diamond and hybrid abrasives at different processing time periods.

Figure 4 exhibits the roughness of sapphire substrate polished by diamond and hybrid abrasives at different processing time periods. In Figure 4, under the same processing conditions, the roughness of sapphire substrate surface polished by hybrid particles is 0.2 nm smaller than these polished by diamond particles. Meanwhile, for the diamond abrasives, the standard deviation of Ra increases when the processing time is 120 min. Then, the Ra increases as the polishing process proceeds. For the hybrid abrasives, the mean value and standard deviation of Ra continue to decrease in 180 min of processing time. So, a higher and more uniform surface quality than that of diamond abrasives can be achieved by using the hybrid abrasives under the same processing conditions.

**Figure 5:**
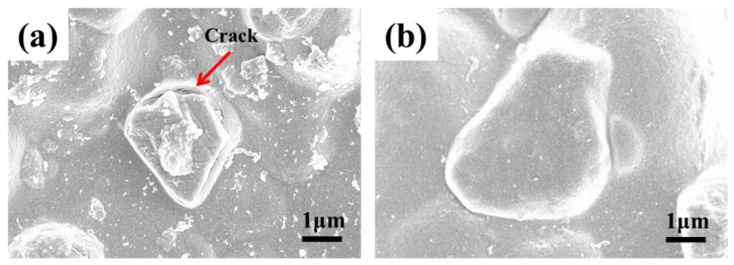

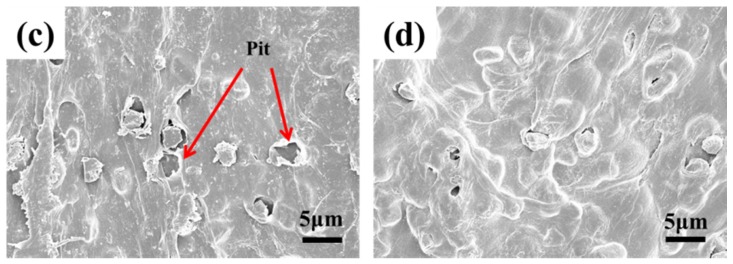
Combination of diamond (**a**,**c**) and hybrid (**b**,**d**) abrasives with the matrix before (**a**,**b**) and after (**c**,**d**) polishing.

Figure 5 shows the binding condition of the diamond and hybrid particles with the matrix before polishing, and the shedding condition of the diamond and hybrid particles from the polishing pad after polishing. Some cracks could be clearly observed at the combination of matrix and diamond particles before polishing (Figure 5a). By contrast, hybrid particle was tightly wrapped in the matrix before polishing (Figure 5b). In addition, the number of hybrid particle residues in the matrix after polishing was significantly higher than that of diamond particles, as shown in Figure 5c,d. All the results could be inferred that the presence of porous and abundant –OH in the akageneite coating are conductive to enhancing the binding force between the matrix and abrasives. This feature is attributed to the tunnel structure and material properties of akageneite. The enhanced binding force can reduce the amount of abrasive grains shedding from polishing film during processing, as well as to prolong the effective processing time of the polishing pad. Moreover, the MRR of sapphire wafer polished by hybrid abrasives is similar to that of diamond abrasives, which is around 0.28 nm/min. For hybrid abrasives, although the coating with low hardness will weaken their material removal ability, a greater number of efficient particles in the polishing pad can make up for this defect to some extent during polishing.

**Figure 6:**
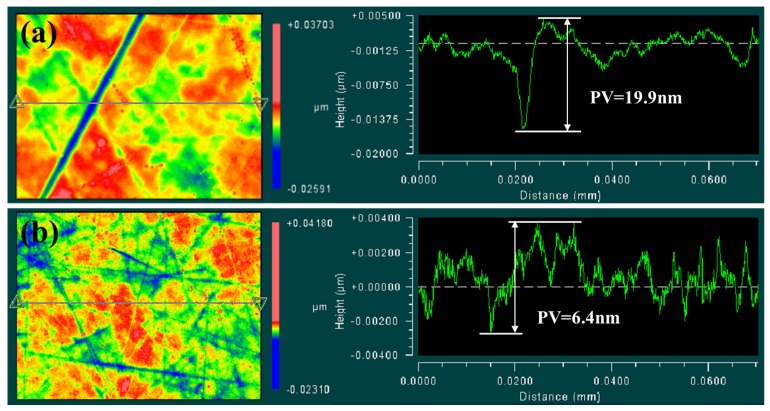
Morphologies and profile curves of the sapphire substrate polished by diamond (**a**) and hybrid (**b**) abrasives.

On the other hand, the morphologies and profile curves of the sapphire substrate polished by diamond and hybrid abrasives were characterized by 3D optical interferometry profiler. As shown in Figure 6, due to the high hardness and sharp edges, the scratches caused by diamond particles are very deep. By contrast, the scratches caused by hybrid particles are very small. Meanwhile, the average value of PV (peak-to-valley) is 17.8 nm by diamond particles, and 7.5 nm by hybrid particles. PV value which mainly indicates the depth of scratch is significantly decreased by using hybrid particles. This phenomenon could be explained by the fact that the core/shell structured hybrid particles exhibit a lower elastic modulus and hardness than the diamond particles. When considering the deformation of the particle, the depth of the particle into the substrate surface can be predicted as [16,28]

(4) $$\delta ={\left(\frac{9F^{2}}{8DE_{\mathrm{sw}}^{2}}\right)}^{\frac{1}{3}}$$

(5) $$\frac{1}{E_{\mathrm{sw}}}=\frac{1-v_{s}^{2}}{E_{s}}+\frac{1-v_{w}^{2}}{E_{w}}$$

(6) $$\delta_{W}=D-\delta -\delta_{p}=D-\delta \left[1+{\left(\frac{E_{\mathrm{sw}}}{E_{\mathrm{sp}}}\right)}^{\frac{3}{2}}\right]$$

where δ is the deformation of the abrasive particle; *F* is the polishing pressure; *D* is the diameter of the particle; *E*_sw_ is the Young’s modulus of the particle and wafer pair; δ_w_ is the the indentation depth of the particle into the substrate surface; δ_p_ is the indentation depth of the particle into the polishing pad; *E*_sp_ is the Young’s modulus of the particle and pad pair; *E*_s_ and *v*_s_ are the Young’s modulus and the Poisson’s ratio of the abrasive particle, respectively; *E*_w_ and *v*_w_ are the Young’s modulus and the Poisson’s ratio of the wafer, respectively.

The indentation depth of the particle into the wafer is decreased with the increase of the deformation of the particle. Due to these properties, core/shell structured hybrid particles can decrease the contact stress and increase the contact area between abrasive and wafer; thus, the core/shell structured hybrid particles can decrease the scratch depth. The removal amount of the next process is mainly determined by the depth of the deepest scratch. The achieved surface polished by hybrid particles with shallow and uniform scratches is critical to obtaining an ultra-smooth and defect-free sapphire substrate surface economically and efficiently.

## 4. Conclusions

The core/shell structured diamond/akageneite hybrid particles were prepared via one-step isothermal hydrolyzing under relatively low temperature. The β-FeOOH coating is uniformly formed on diamond particle surface, and the structure of coating is spindle-shaped. The hybrid particles can reduce the scratch depth without compromising the MRR and surface roughness. Compared with pristine diamond, the PV value of sapphire substrate surface after polishing has been decreased 10.3 nm by using hybrid particles. Moreover, the particles exhibited less abrasive shedding during the processing. The hybrid particle significantly improved the polishing performance of SG polishing pad for sapphire substrate. It can be attributed to the β-FeOOH coating which can improve the surface quality and prolong the effective processing time of polishing pad.
